# Why coronavirus survives longer on impermeable than porous surfaces

**DOI:** 10.1063/5.0037924

**Published:** 2021-02-09

**Authors:** Sanghamitro Chatterjee, Janani Srree Murallidharan, Amit Agrawal, Rajneesh Bhardwaj

**Affiliations:** Department of Mechanical Engineering, Indian Institute of Technology Bombay, Mumbai 400076, India

## Abstract

Previous studies reported that the drying time of a respiratory droplet on an impermeable surface along with a residual film left on it is correlated with the coronavirus survival time. Notably, earlier virus titer measurements revealed that the survival time is surprisingly less on porous surfaces such as paper and cloth than that on impermeable surfaces. Previous studies could not capture this distinct aspect of the porous media. We demonstrate how the mass loss of a respiratory droplet and the evaporation mechanism of a thin liquid film are modified for the porous media, which leads to a faster decay of the coronavirus on such media. While diffusion-limited evaporation governs the mass loss from the bulk droplet for the impermeable surface, a much faster capillary imbibition process dominates the mass loss for the porous material. After the bulk droplet vanishes, a thin liquid film remaining on the exposed solid area serves as a medium for the virus survival. However, the thin film evaporates much faster on porous surfaces than on impermeable surfaces. The aforesaid faster film evaporation is attributed to droplet spreading due to the capillary action between the contact line and fibers present on the porous surface and the modified effective wetted area due to the voids of porous materials, which leads to an enhanced disjoining pressure within the film, thereby accelerating the film evaporation. Therefore, the porous materials are less susceptible to virus survival. The findings have been compared with the previous virus titer measurements.

Infectious disease such as COVID-19, caused by SARS-CoV-2 (referred to as *coronavirus* hereafter), is transmitted through respiratory droplets.^1–4^ Apart from airborne infection spread,^5,6^ the virus-laden droplets also form fomite upon falling on a surface,^7–9^ which serves as a source for infection spread. Therefore, the use of face mask and sanitization of surfaces of daily use have been recommended by the WHO.^10^ It is, therefore, important to understand the mechanism of virus survival on different surfaces. Previous investigations looked into the survival on different surfaces by depositing a 5 *µ*l virus-laden droplet (dose ∼7 · 8 log unit of TCID_50_ per ml) and monitoring the decay in the titer value with time.^11,12^ On the other hand, since the droplet serves as a medium for virus survival, the infectivity of the virus is connected to an extent to the droplet lifetime, which led to research on droplet evaporation on different surfaces and the factors affecting it.^13–15^ For instance, the decay in the infectivity of 19 different viruses upon drying of virus-laden droplets on glass slides under given ambient temperature and humidity conditions was experimentally studied.^13^ The importance of evaporation dynamics in studying the transmission probability, and the survival of enveloped viruses such as coronavirus, was also recognized recently by Mittal *et al.*,^6^ as evaporation has a paramount important role in the eventual fate of a droplet. Additionally, Chaudhuri *et al.*^4^ and Bhardwaj and Agrawal^16^ examined the correlation between the drying time and the growth of infection for the cases of droplets suspended in air and deposited on a surface, respectively. Identifying evaporation as one of the major contributing factors to the virus survival, recent studies have disclosed that the nature of the underlying surface and the ambient conditions (temperature and humidity) play a significant role in determining the droplet drying time and thereby dictating the vulnerability of different surfaces and environmental condition regarding the risk of infection spread.^16,17^ Tailoring wettability can serve as a tool to reduce the risk of infection.^18^ Bhardwaj and Agrawal^16,17^ adapted a model approach to study the evaporation of a sessile droplet and a residual thin film on solid surfaces. They established that by considering a surrogate droplet of pure water (without considering the presence of virus and the associated shear stress, and biological solutes contained in saliva/mucus droplets), the model could yield a reasonable qualitative explanation and a comparative understanding of coronavirus survival on different surfaces under different environmental conditions. The associated risk factors of COVID-19 spread were also assessed with reasonable fidelity. Particularly, it was shown that at a later stage, the drying time scale of the residual thin film is correlated with the decay time scale of the virus titer values.^17^ Therefore, understanding droplet and residual thin film evaporation is important in the context of virus survival.

While the previous studies^16,17^ successfully demonstrated the evaporation rate on flat surfaces, a tie between the drying time and the virus survival found from the titer measurements was established; knowledge on virus survival on porous surfaces, e.g., paper and cloth, has not been disseminated yet. Porosity has been found to be a major factor in determining the inactivation rates of influenza-type viruses.^14^ The importance further arises from the fact that the investigations by measuring virus titer^11,12^ had revealed that porous materials such as wood, cloth, and paper are significantly less favorable for virus survival, i.e., the survival time of the coronavirus on these surfaces is surprisingly less as compared to impermeable surfaces, such as glass, stainless steel, and plastic. Therefore, the goal of the present work is to shed light on the reason behind the significantly less virus survival time on porous surfaces as compared to impermeable surfaces by raising the following question: What is the influence of porosity in modifying the evaporation mechanism on porous materials? In order to reach the research goal, first, experiments on droplet evaporation on both porous and non-porous surfaces were performed by employing 1 *μ*l pure aqueous droplets to gain insights into how differently a droplet interacts with porous vs impermeable surfaces. Figure 1 shows a schematic of the problem considered. The distinction of the present work lies in the fact that in contrast to the case of flat, impermeable surfaces, porous surfaces exhibit a strong capillary imbibition effect that plays a key role in determining the decay of droplet volume with time as the process of imbibition is significantly faster than that of evaporation; this sets the initial condition for the later stage when a thin liquid film is left on the solid parts of the surface after the evaporation of the bulk droplet. Second, at the later stage, Bhardwaj and Agrawal^17^ showed that the disjoining pressure-driven evaporation of the residual thin film is much slower, implying the drying time scale closer to that of virus titer decay. However, their study was limited to impermeable surfaces. The mechanism of evaporation of the residual thin film for the case of porous geometry is still unknown, which is the subject of investigation of the present work. The earlier model has been extended herein to gain insights into the modified evaporation mass flux profile on porous geometry. Briefly, the present work discloses a distinct aspect of liquid mass loss from porous surfaces—imbibition followed by drying of a residual thin film with a modified evaporative mass flux profile. The findings demonstrate reasonably well the reason behind the less survival of the coronavirus on porous surfaces found in the titer measurements. The insights gained from the present study are of fundamental importance as well as useful to demonstrate the extent of the safety of different objects of daily use, thereby raising public awareness.

**FIG. 1.**
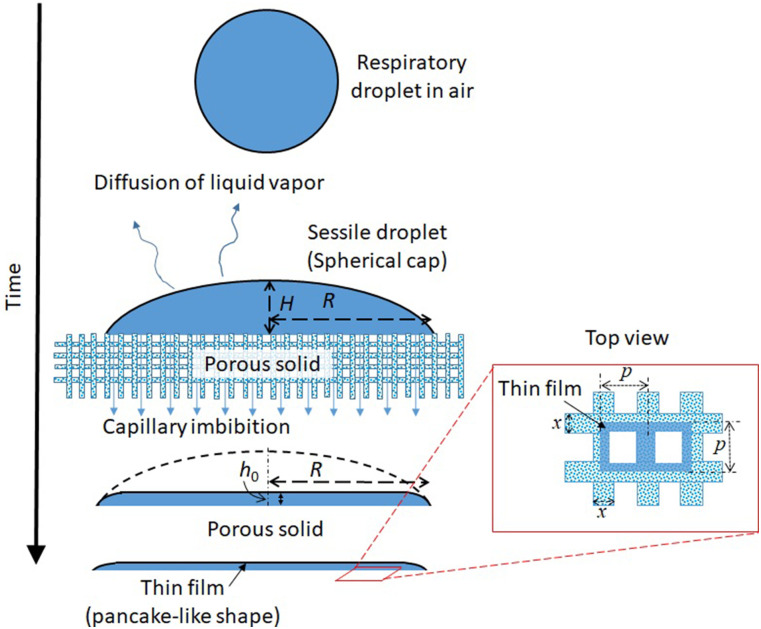
Schematic of the problem considered in the present work. The inset shows the geometry of porous fibers.

The drying of 1 *μ*l droplets of pure water placed on impermeable and porous surfaces was recorded using a high-resolution camera, and optical microscopy was employed to characterize the porous surfaces. The materials used in the present experiments have been carefully chosen to enable the authors to feasibly compare the findings with the previous virus titer measurements. While plane glass, stainless steel, and plastic were used as the impermeable surfaces, paper and cloth were chosen for porous materials. The detailed experimental procedure, data acquisition, and data analysis along with the sample preparation and cleaning procedure, and the surface characterization are provided in Sec. S1 of the supplementary material. In this Letter, we define the “*virus survival time*” as the time duration in which the virus titer decayed to an *undetectable* value in the previously reported measurements.^11,12^ This time scale has been used herein for comparison with the present findings.

First, the experimental results on the evaporation of the aqueous droplet on smooth surfaces are presented. A droplet is gently deposited on the surface under investigation, and the initial time (*t* = 0) is set when contact between the droplet and the substrate surface is established, and subsequently, the droplet assumes an equilibrium spherical cap shape. Figure 2 (Multimedia view) depicts a representative result of the temporal evolution of droplet geometry as it evaporates on a glass substrate. Similar experiments were performed by using plastic and stainless steel as substrates, and the results are shown in Fig. S3 of the supplementary material. While on glass and plastic, the evaporation occurs mostly in the constant contact area mode, in the case of stainless steel, the dynamics of the triple-phase contact line is initially in the constant contact area mode, and a mixed-mode comprising both constant contact area and constant contact angle is observed at the later stages of evaporation. These observations are consistent with the previous observations.^19,20^ It is well understood that the principle governing mechanism of droplet mass loss from a solid, impermeable surface is the diffusion-limited evaporation.^21^

**FIG. 2.**
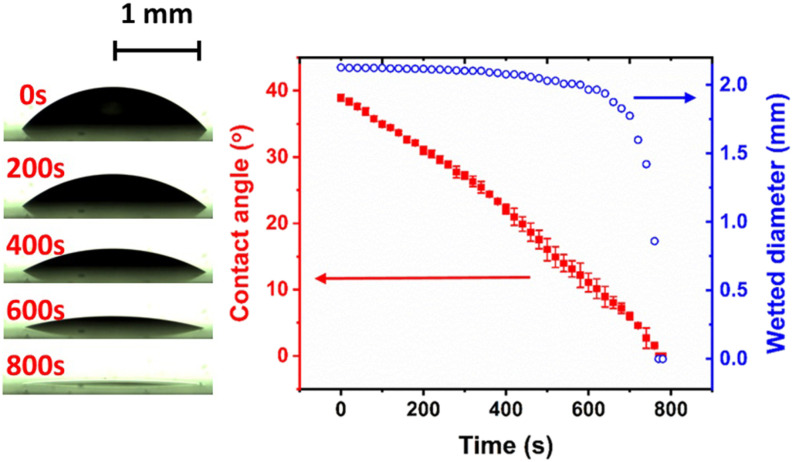
Temporal evolution of a 1 *µ*l aqueous droplet as it evaporates on glass. Left column shows the droplet shapes at different time frames. Similar experiments were performed on plastic and stainless steel, and the results are shown in Fig. S3 of the supplementary material. Movies depicting the evaporation of the droplet on glass, plastic, and stainless steel substrates, from which the data are extracted. Multimedia view: https://doi.org/10.1063/5.0037924.110.1063/5.0037924.1

The bulk droplet lifetimes on glass, plastic, and stainless steel are about 800 s, 1400 s, and 1600 s, respectively. It is well documented from both experiments and the diffusion-limited model^19,21^ that the droplet lifetime is primarily governed by the wettability, which is consistent with the present observations; the drying time increases with reducing wettability (larger contact angle)—from glass to stainless steel. From the virus titer measurements,^12^ the virus survival times on these surfaces were found to be 4 days, 7 days, and 7 days, respectively. In this way, the ratio of virus survival time in titer experiments (glass:plastic:stainless steel = 4:7:7) agrees qualitatively with the present experiments (glass:plastic:stainless steel = 4:7:8). The correlation between the droplet evaporation rate and the virus survival time on different surfaces is thus realized, which was earlier envisioned by a diffusion-limited evaporation model.^16^ However, virus titer measurements had revealed that the coronavirus can survive on a given surface for several hours/days. This fact was reasoned by a model involving the disjoining (film) pressure-driven slower evaporation of a thin liquid film left behind the evaporated droplet.^17^ Further details of thin film evaporation dynamics and its modification for porous media will be discussed later in this Letter.

Second, how differently a droplet interacts with a porous surface as compared to impermeable surface is investigated. Figure 3 (Multimedia view) shows both qualitatively and quantitatively the temporal variation of droplet geometry on porous surfaces under investigation. The general features of droplet behavior are as follows. The droplet is first deposited on the substrate surface and initially assumes a spherical cap shape. Thereafter, it spreads over the surface, which is attributed to the adhesion between the horizontally oriented fibers and the liquid near the contact line region.^22^ The contact angle decays to almost zero, which is caused by both spreading and liquid imbibition through the pores.^23,24^ Thereafter, a patch of liquid appears, which is visible from the top. The wetted patch, retaining its area, subsequently decays and eventually disappears after a certain time. The consequence of the spreading and imbibition will be discussed later in this Letter. As depicted in Figs. 3(a) and 3(b) (Multimedia view), on paper, the spreading starts and the contact angle decays to an undetectable value in initial ∼25 s. The continuous decrease in the contact angle (*θ*) is shown quantitatively in Fig. 3(d-i). For cloth, this initial stage occurs in a much shorter duration (∼2.5 s). The data can be well fitted with an exponential decay [*θ* = *θ*_0_ exp(−*t*/t^*^)] with the corresponding characteristic time t^*^ = 8.47 s and 0.815 s for paper and cloth respectively. At the later stage, the liquid is hidden within the fibrous surface, making the side visualization impossible. However, top views show that a liquid patch appears, which subsequently gets fainted [cf. Fig. 3(b) (Multimedia view)], keeping the wetted area the same, and is eventually disappeared at t ∼ 155 s for paper. On cloth, the wetted patch forms earlier [cf. Fig. 3(c) (Multimedia view)]. Interestingly, the wetted patch lasts much longer on cloth (∼300 s) as compared to that on paper (∼155 s). A comparative study of temporal droplet spreading on paper vs cloth is presented in Fig. 3(d-ii). The wetted diameter is normalized to its initial value, and the time is normalized to the respective t^*^ obtained from Fig. 3(d-i). The data are plotted until the time the wetted patch remained detectable by using the camera. It is interestingly noted that the spreading is much larger on paper than that on cloth, while the disappearance time of the wetted patch is much shorter on paper than cloth. Both the data in Fig. 3(d-ii) can be well fitted by the following relation: *y* = *A* + *b*(1 − exp(−*x*/*p*)). The values of the fitted parameters *θ*_0_, t^*^, *A*, *b*, and *p* are listed in Table S1 of the supplementary material.

**FIG. 3.**
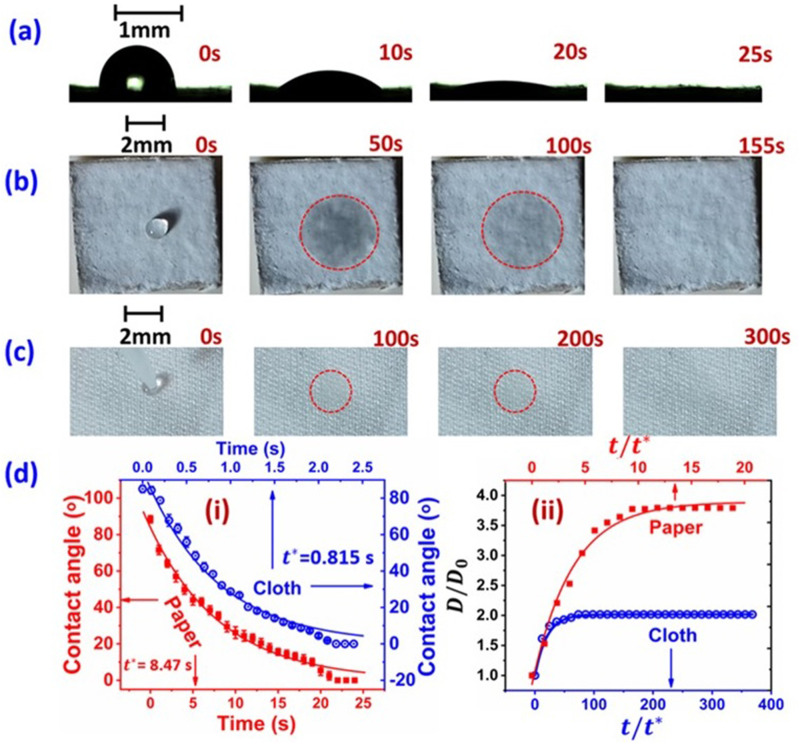
Droplet spreading and temporal evolution of the droplet geometry on porous media. (a) Paper, side view; (b) paper, top view; and (c) cloth, top view. The red dotted circles indicate the wetted diameter of the liquid patch left after complete spreading of the droplet, i.e., when the contact angle reaches to zero. (d-i) Temporal variation of contact angle on paper and cloth. (d-ii) Temporal variation of wetted diameter on paper and cloth derived from the top views. Movies depicting the temporal variation of droplet geometry on porous surfaces, from which the data have been extracted. Multimedia view: https://doi.org/10.1063/5.0037924.210.1063/5.0037924.2

A porous media is characterized by porosity *φ*, which is the ratio between the void volume (*V*_v*oid*_) and the total volume (*V*_*tot*_) of the media. The characteristic distinction of porous media over flat surfaces lies in the fact that capillary imbibition plays a dominant role in draining the liquid out from the top surface. It is assumed for simplicity that the porous structure is filled vertically, i.e., there is no radial flow within the porous material, and that filling at a particular radius starts when the contact line reaches that radius. In real systems, the radial flow will occur; however, its effects are small for isotropic pore structures, as over the lifetime of the drop, the radius of contact between the drop and the substrate is large compared with the penetration distance.^25^ Assuming the porous medium to be an array of cylindrical capillary tubes, the penetration length (*l*) in a single pore of the liquid plug at a generic time (*t*) is given by Washburn’s equation,^26^

$$l=\sqrt{\frac{r_{p}\gamma \;cos\;\theta }{4\mu }t},$$ (1)

where *r*_*p*_, *γ*, *μ*, and *θ* are the pore radius, liquid surface tension, liquid viscosity, and the intrinsic contact angle of the material of the porous medium, respectively. Hence, the volume drained by a single pore at time *t* is

$$V=\pi r_{p}^{2}l=\pi r_{p}^{2}\sqrt{\frac{r_{p}\gamma \;cos\;\theta }{4\mu }t}.$$ (2)

If *δ* is the area fraction of the pores and *R* is the instantaneous radius of the wetted area, the number of wetted pores is $n=\pi R^{2}\delta /\pi r_{p}^{2}$. Hence, the total volume drained (*V*_*d*_) by the wetted pores at *t* [by virtue of Eq. (2)] is

$$V_{d}=nV=\pi R^{2}\delta \sqrt{\frac{r_{p}\gamma \cos\theta }{4\mu }t}.$$ (3)

Equation (3) shows that the *V*_*d*_ scales as $\sqrt{t}$ and is also dependent on pore size (*r*_*p*_), pore surface area fraction (*δ*), and the intrinsic wettability of the material involved. It shows that for the materials with larger wettability (smaller *θ*), more imbibition occurs, which is consistent with the previous studies,^23,24^ wherein the authors reported that for a wettable porous media (*θ* = 30°, 45° considered therein), the imbibition takes place continuously until the top surface is completely dry. This is consistent with the present experimental observations; a continuous drainage is observed until the upper surface is left completely dry. Furthermore, Fig. 4(a) shows that the temporal decay of droplet volume exhibits a linear nature for glass,^19,21^ while for paper, the trend is non-linear and much faster than glass. Hence, for porous media, the droplet mass loss is mainly governed by imbibition, as opposed to impermeable media, where the diffusion-limited evaporation dominates the mass loss. To further address this issue, the drained volume [*V*_*d*_(*t*)] is plotted against the square root of time ($\sqrt{t}$) for the experimental data of paper in Fig. 4(b). Since the droplet remains in a spherical cap shape, throughout the spreading time, the instantaneous volume of the droplet [*V*(*t*)] is related to the instantaneous droplet radius [*R*(*t*)], the instantaneous droplet height [*H*(*t*)], and the instantaneous contact angle [*θ*(*t*)] as follows:

$$V\left(t\right)=\frac{\pi H\left(t\right)}{6}\left[{\left(3R\left(t\right)\right)}^{2}+{\left(H\left(t\right)\right)}^{2}\right],\theta \left(t\right)=2\tan^{-1}\frac{H\left(t\right)}{R\left(t\right)}.$$ (4)

Given that *V*_0_ is the initial droplet volume, *V*_*d*_(*t*) can be determined as

$$V_{d}\left(t\right)=V_{0}-V\left(t\right).$$ (5)

**FIG. 4.**
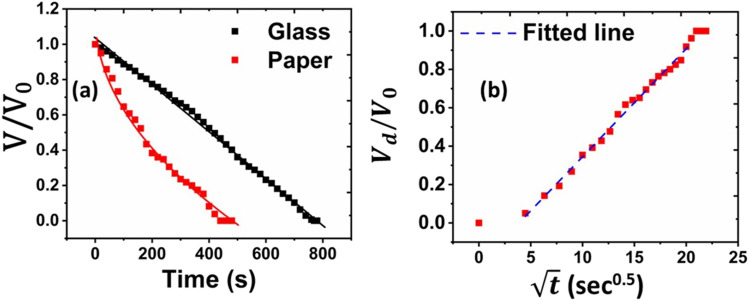
(a) Temporal decay of normalized droplet volume for glass and paper and (b) drained volume vs square root of time plot and linear fitting for paper.

Figure 4(b) shows that the variation of *V*_*d*_(*t*) and $\sqrt{t}$ can be well fitted by a straight line with an adjusted R-square value of ∼0.99, depicting that the present measurements are, in principle, governed by Eq. (3). Therefore, it is concluded that at the initial stage, liquid imbibition plays a dominant role in the loss of droplet volume. This is in contrast to the case of flat impermeable surfaces, where diffusion-limited evaporation dominates the mass loss of the droplet. It is also seen that liquid imbibition is a much faster process as compared to evaporation. After the drainage of liquid by the capillary imbibition process, a thin liquid film remains on the solid parts of the porous media.

Third, we explain the mechanism of thin liquid film evaporation from porous media and its connection to the survival of the coronavirus. However, at first, the evaporation behavior of a thin liquid film and the corresponding governing equations on flat surfaces will be presented. Thereafter, the attention will be shifted to the evaporation of the thin liquid film on porous surfaces, and the modification of the associated governing equations will be explained. This systematic approach would be helpful to understand the contrast between the mechanisms of thin film evaporation on impermeable and porous surfaces, thereby demonstrating the virus survival time on them. The time variation of thickness (*h*) of the evaporating thin film on a smooth solid surface is given by^17^

$$\frac{dh}{dt}=\frac{J}{\rho_{L}},$$ (6)

where *ρ*_*L*_ is the liquid density (=1000 kg/m^3^ for water) and the evaporation mass flux *J* is given by

$$J=\frac{\rho_{V}}{\rho_{L}\sqrt{2\pi RT_{amb}}}\left[\frac{A_{H}}{6\pi h^{3}}-\frac{\gamma h}{R^{2}}\right],$$ (7)

where *A*_*H*_, *γ*, and *R* are the Hamaker constant of interaction between liquid–vapor and solid–liquid interfaces, the surface tension of the liquid (0.072 N/m for water), and wetted radius, respectively. In Eq. (7), R (=461.5 J/kg K) is the specific gas constant for water vapor, *ρ*_*V*_ (=0.023 kg/m^3^) is the density of water vapor at ambient, and *T*_*amb*_ (=298 K) is the ambient temperature. Using the values of R, *ρ*_*V*_, and *T*_*amb*_, the prefactor outside the parentheses of Eq. (7) can be calculated as *a* = 2.47 × 10^−11^ SI units. The first term within the parentheses of Eq. (7) represents the disjoining pressure *P*(*h*) within the film. Note that since *P*(*h*) is a representative of the solid–liquid interfacial energy, the liquid thin film would evaporate slower on surfaces having a lower surface free energy (higher contact angle). In the present experiments (cf. Fig. 2), the contact angles of water droplets on glass, plastic, and stainless steel were found to be ∼39°, 86°, and 90°, respectively, which indicates that the surface energy is decreased progressively from glass to stainless steel. This implication is consistent with the film evaporation rate^17^ as well as the survival of the coronavirus observed in the titer measurements^11,12^ on surfaces with varying wettability. The second term within the parentheses of Eq. (7) is the Laplace pressure term. It was previously shown^17^ that the Laplace pressure is one order of magnitude less than the disjoining pressure and therefore can be ignored in Eq. (7). Furthermore, a detailed demonstration of the negligible contribution of the Laplace pressure term to Eqs. (6) and (7) is provided in Fig. S4 of the supplementary material. Hence, neglecting the Laplace pressure term in Eq. (7) and then integrating Eq. (6) with respect to time (*t*) give *h* as a function of *t* as follows:

$$h^{4}=h_{0}^{4}+\frac{4aA_{H}}{6\pi }t.$$ (8)

Figure 5 shows the variation of *h* with *t* for glass, wherein the initial film thickness (*h*_0_) has been taken as 400 nm. This value has been used in our previous study on impermeable surfaces, and a good agreement between the decrease in film volume and virus titer was found.^17^ Here, *A*_*H*_ = −1.3 × 10^−20^ J (cf. Sec. S2 v of the supplementary material). Figure 5 depicts that the exact solution of the governing equation [Eq. (8)] returns a film lifetime of ∼104 h. This time scale is consistent with the virus survival time on glass (∼4 days = 96 h) found in titer measurements. Thus, the correlation between the virus survival time and the film lifetime is realized with reasonable fidelity.

**FIG. 5.**
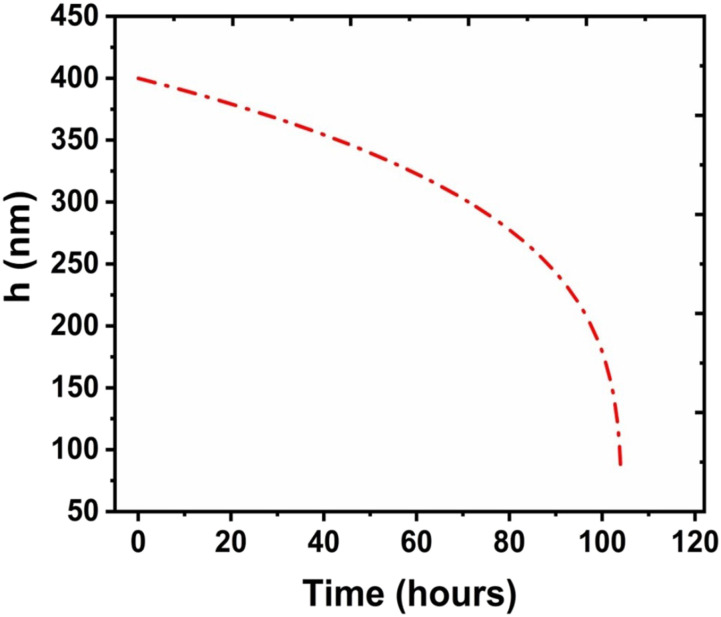
Variation of thin liquid film thickness with time on the glass substrate computed from the model.

Why the coronavirus survives for a less duration on porous media? As mentioned earlier, the virus titer measurements have revealed a significantly less survival time on porous media (∼2 days on cloth and just ∼3 h on paper^12^). The previous model^17^ developed for an impermeable surface is not appropriate to explain the survival time on the porous surface. It is, therefore, important to carry out a close investigation on the modified mechanism of thin film evaporation on porous surfaces and to understand how the decay of the virus is accelerated in the case of porous surfaces. The wettability would modify *J* in a similar fashion for both porous and impermeable surfaces, as it is dictated by *A*_*H*_ (surface energy) in Eq. (7). Hence, the key factor that differentiates a porous material from an impermeable one is the geometry. Therefore, to look into the effect of modified geometry, an energy argument similar to that of Wenzel^27^ is considered herein. The equilibrium between the different interfacial energies, namely, the liquid–vapor (*γ*_*LV*_), solid–vapor (*γ*_*SV*_), and solid–liquid (*γ*_*SL*_) interfacial energies in terms of contact angle (*θ*), is given by the classical Young’s equation,^27^

$$\gamma_{LV}\cos\theta =\left(\gamma_{SV}-\gamma_{SL}\right)=E_{SL},$$ (9)

where *E*_*SL*_ is the energy required to form a unit area of the solid–liquid interface.^28^ In the case of rough surfaces, the Wenzel argument states that as the liquid front advances against the solid surface, each solid–vapor interface is replaced by an equal amount of solid–liquid interface (cf. Fig. S5 of the supplementary material), and *E*_*SL*_ is enhanced by the surface area factor (*r*_*a*_), which is the ratio between the actual area of the rough surface and the projected area.^27,28^ This argument can be extended for the case of thin film as follows. For the case of a film covered surface (cf. Fig. S5 of the supplementary material), the modified surface energy (*γ*_*SV*_′) reads as^29^

$$\gamma_{SV}^{\prime }=\gamma_{SL}+\gamma +E\left(h\right),$$ (10)

where E(h) is the excess energy of the film, which is the derivative of the disjoining (film) pressure [*P*(*h*) = *A*_*H*_/6*πh*^3^]. Hence, for the case of rough surfaces, E(h) [or *P*(*h*)] would be enhanced by a factor of *r*_*a*_, by virtue of the enhancement of the term [*E*_*SL*_′ = (*γ*_*SV*_′ − *γ*_*SL*_)] by the factor of *r*_*a*_ (cf. Fig. S5 of the supplementary material). The same argument is hereby extended to porous media as discussed below.

 For the case of porous media, let us assume that the energy enhancement factor due to the modified surface exposure area is *ϕ*, which stems from two distinct physical features of the porous media, as observed in the experiment [cf. Fig. 3 (Multimedia view)]. First, the droplet spreads over the surface, which is attributed to the capillary action between the fibers and the droplet edge. Since the fibers are oriented horizontally, adhesion between the solid and the liquid causes the droplet edge to traverse through the pathways of the fibers.^22^ Hence, if *ϕ*_1_ is assumed to be the energy enhancement due to spreading, then $\phi_{1}=\left(R_{2}^{2}/R_{1}^{2}\right)$, where *R*_2_ is the final wetted radius after complete spread and *R*_1_ is the initial wetted radius before spreading, i.e., when the droplet rests on the surface and assumes an equilibrium spherical cap shape. From the experiments, *ϕ*_1_ = 25 and 4 for paper and cloth, respectively. The second contribution to *ϕ* is stemmed from the void areas present on a porous surface, which is assumed to be *ϕ*_2_. In order to calculate *ϕ*_2_, a specific geometry similar to that of Fig. 1 in Ref. 30 is considered herein, as this is the typical geometrical feature for woven fabrics [cf. Fig. S2(a) of the supplementary material].^31^ A typical geometry considered in the present calculations is shown in the inset of Fig. 1.

Let us assume that the fabrics are having a square cross section of area, *x* × *x*, and they are woven such that the voids among them are cubes (cf. the inset in Fig. 1). If the pitch of the fibers, i.e., center to center distance between two consecutive fibers is *p*, the edge of the void cube is happened to be *p* − *x*. A unit cell of the fabric with cubic volume *p*^3^ consists of both solid (fiber) and void. The solid volume contained within the cell can be estimated using geometry and is expressed as follows:

$$V_{solid}=12\left(x/2\right)\left(x/2\right)\left(p-x\right)+8{\left(x/2\right)}^{3}=3x^{2}\left(p-x\right)+x^{3}.$$ (11)

Hence, porosity is given by

$$\varphi =1-\frac{V_{solid}}{V_{total}}=2r^{3}-3r^{2}+1,$$ (12)

where *r* = *x*/*p*. The area of the top surface of the cube is *A*_*total*_ = *p*^2^. The wetted area contained within the top face of the cube is expressed as

$$A_{wetted}=4\left(x/2\right)\left(p-x\right)+4{\left(x/2\right)}^{2}=x\left(2p-x\right).$$ (13)

Hence, the wetted area fraction is given by

$$\phi_{2}=\frac{A_{wetted}}{A_{total}}=r\left(2-r\right).$$ (14)

Thus, *r* is a varying parameter from which *φ* and the corresponding *ϕ*_2_ can be computed. Figure 6(a) shows the variation of *φ* and *ϕ*_2_ with respect to *r*, while Fig. 6(b) depicts the variation of *ϕ*_2_ with respect to *φ*. It is noticed that the limiting conditions (*ϕ*_2_ → 1 as *r* or *φ* → 0 and *ϕ*_2_ → 0 as *r* or *φ* → 1) are satisfied by the calculations. The porosity values as indicated in Fig. 6(b) have been taken from Refs. 30–33. In the present analysis, *ϕ*_2_ has been chosen from the corresponding *φ* values in Fig. 6(b). The effective energy enhancement factor, *ϕ*, thus becomes *ϕ* = *ϕ*_1_*ϕ*_2_. Therefore, for porous surfaces, the modified *J* profile is expressed as follows: *J*_*mod*_ = *ϕJ* by virtue of the enhancement of *P*(*h*) by *ϕ*, and the governing equation (6) for the evaporating thin liquid film is modified as

$$\frac{dh}{dt}=\frac{J_{mod}}{\rho_{L}}.$$ (15)

**FIG. 6.**
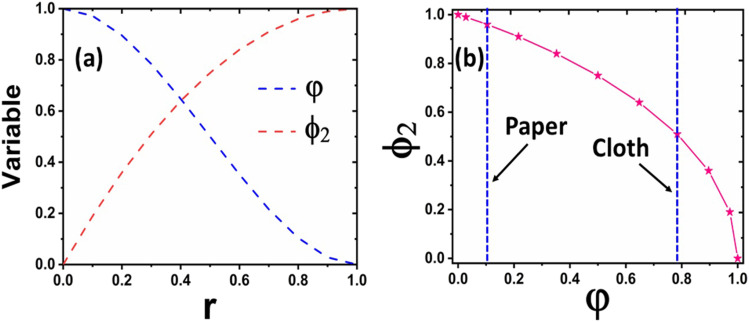
(a) Variation of *φ* and *ϕ*_2_ with *r* and (b) variation of surface area fraction *ϕ*_2_ with porosity *φ*.

Equation (15) can be integrated in the same way as was performed to derive Eq. (8) from Eqs. (6) and (7) for obtaining *h* as a function of *t*. Figures 7(a) and 7(b) show *h* vs *t* curves for cloth and paper, respectively. *A*_*H*_ = −9.8 × 10^−21^ J [cf. Sec. S2 v of the supplementary material]. From Fig. 7(a), the thin film lifetime is found to be ∼60 h on cloth, which is in reasonable agreement with the coronavirus survival time found from the titer measurements on cloth (∼2 days = 48 h). From Fig. 7(b), the thin film lifetime is found to be ∼5 h on paper, while the coronavirus survival time found from the titer measurements was ∼3 h. Hence, it may be concluded that the present analysis could capture the virus survival time on cloth with reasonable accuracy, while for paper, it is reasonably consistent with the extent of order of magnitude. Therefore, the present model explains the essential physics behind the less virus survival time on the porous surfaces. The quantitative discrepancy found for the case of paper may be attributed to the fact that the specific geometry considered herein to calculate the solid area fraction on porous surfaces (cf. Fig. 1, inset) is closest to that of woven cloths [cf. Fig. S2(a) of the supplementary material].^30,31^ For paper, the surface is more irregular, and the geometry is different from that of cloth [cf. Fig. S2(b) of the supplementary material].^32,33^ Therefore, because of the specific geometry consideration, the outputs of the present analysis are closest to the virus survival time of cloth, while for paper, a qualitative agreement is obtained. Hence, it can be asserted that the combined information gained from Fig. 7 essentially demonstrates why coronavirus was found to survive surprisingly less on porous surfaces, as compared to flat solid surfaces; it is the fiber-droplet capillary action-driven droplet spreading and the exposed solid area modification due to the voids, which causes an enhanced film pressure within the thin liquid film, thereby accelerating the film evaporation. Some limitations of the present analysis can be addressed in the future. Pure water has been considered herein, while saliva/mucus respiratory droplets containing biological solutes may exhibit a non-Newtonian behavior and different surface tensions and viscosities, which may influence the drying time. However, as outlined previously,^17^ the uncertainty due to the aforesaid approximation is not significant (∼25%), and the analysis can explain the virus survival qualitatively and comparatively with a reasonable fidelity, highlighted earlier in the Introduction. Furthermore, modifications in the initial contact angle and *A*_*H*_ should be accounted for while dealing with a pre-wetted surface,^29^ which is beyond the scope of the present work.

**FIG. 7.**
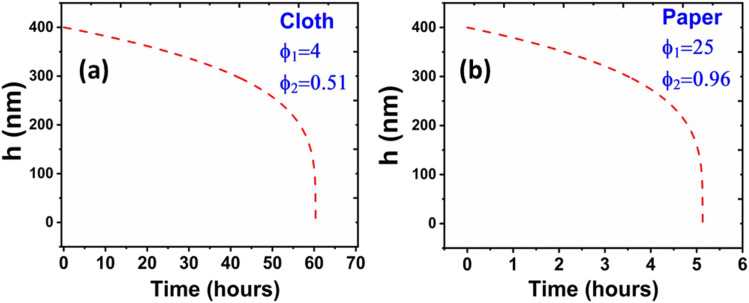
Variation of film thickness with time for (a) cloth and for (b) paper found from the model.

Finally, the relevance of the present results in the context of the spread of COVID-19 via fomite is discussed. The usage of cardboard (a porous material) boxes is common by the e-commerce companies, and the present analysis indicates that a less survival duration on porous surfaces implies a reduced risk in a warehouse or package sorting centers. A much less survival on the paper further indicates significantly reduced risk in a classroom, particularly relevant information to the policymakers while considering the re-opening of schools during the pandemic. Similarly, the risks associated with the spread in a garment factory and cloth outlets in shopping malls are much less, as previously thought. We emphasize that the present study focuses on the fomite route of transmission; the airborne transmission should be further properly accounted for to assess the total risk of COVID-19 spread in the above-mentioned examples.

In conclusion, one of the contributing reasons behind the less survival time of coronavirus on porous surfaces as compared to that on impermeable surfaces has been deciphered herein by analyzing the respiratory droplet evaporation mechanism. While for impermeable surfaces, the diffusion-limited evaporation dominates the mass loss from the bulk droplet, for porous materials, the capillary imbibition dominates the process. The latter is a much faster process than the former. After the bulk droplet vanishes, a thin liquid film remains over the exposed solid area, which serves as a medium for virus survival, and its evaporation rate is mainly controlled by the disjoining pressure. However, the thin film evaporates much faster on porous surfaces than on impermeable surfaces. The faster film evaporation rate on the former is attributed to increased disjoining pressure, triggered by an enhanced capillary-driven droplet spreading and the exposed area modification due to the voids.

See the supplementary material for a detailed experimental procedure, supporting results, schematics, and calculations.

## Data Availability

The data that support the findings of this study are available from the corresponding author upon reasonable request.
